# Assessing spatial variability of soil organic carbon and total nitrogen in eroded hilly region of subtropical China

**DOI:** 10.1371/journal.pone.0244322

**Published:** 2020-12-21

**Authors:** Jing Zhang, Miao Zhang, Shaoyan Huang, Xuan Zha

**Affiliations:** 1 College of Geographical Sciences, Fujian Normal University, Fuzhou, Fujian, China; 2 Key Laboratory for Subtropical Mountain Ecology (Ministry of Science and Technology and Fujian Province Funded), Fuzhou, Fujian, China; 3 Department of Ecological Environment, Fujian Provincial Investigation Design & Research Institute Of Water Conservancy & Hydropower, Fuzhou, Fujian, China; Shandong University, CHINA

## Abstract

The hilly red soil region of southern China suffers from severe soil erosion that has led to soil degradation and loss of soil nutrients. Estimating the content and spatial variability of soil organic carbon (SOC) and soil total nitrogen (STN) and assessing the influence of topography and land-use type on SOC and STN after years of soil erosion control are important for vegetation restoration and ecological reconstruction. A total of 375 topsoil samples were collected from Changting County, and their SOC and STN distributions were studied by using descriptive statistics and geostatistical methods. Elevation, slope, aspect and land-use type were selected to investigate the impacts of natural and human factors on the spatial heterogeneity of SOC and STN. The mean SOC and STN concentrations were 15.85 and 0.98 g kg^-1^ with moderate spatial variations, respectively. SOC and STN exhibited relatively uniform distributions that decreased gradually from the outside parts to the center of the study area. The SOC and STN contents in the study area were still at moderate and low levels after years of erosion control, which suggests that soil nutrient improvement is a slow process. The lowest SOC and STN values were at lower elevations in the center of Changting County. The results indicated that the SOC and STN contents increased most significantly with elevation and slope due to the influence of topography on the regional natural environment and soil erosion in the eroded hilly region. No significant variations were observed among different slope directions and land-use types.

## Introduction

Soil organic carbon (SOC) and total nitrogen (STN) are the major indexes used to estimate soil fertility and quality [1, 2]. SOC plays a vital role in mitigating global climate change, and alleviates land degradation and enhancs crop production and food security [3–5]. STN also plays an important role in generating and enhancing soil productivity in terrestrial ecosystems [6, 7]. As dynamic components of terrestrial ecosystems, SOC and STN are characterized by high spatial heterogeneity with complex physical, chemical, and biological processes [4].Thus, knowledge of the spatial variations of SOC and STN is necessary for evaluating ecosystem productivity [8].

The spatial heterogeneity of soil nutrients has been a research focus in the soil and environmental sciences for decades [9–12]. Owing to the high cost of sample collection and analysis, it is difficult to acquire regional details of soil nutrient distributions by large-scale sampling [13], and many reports on the spatial variability of SOC and STN have been carried out at field scales [1, 8]. Precisely predicting soil nutrient contents at regional scales is a significant challenge [14]. The development of precise agricultural research and advances in combining geostatistics and geographic information systems (GIS) have strengthened soil nutrient research [15] and have enabled large-scale estimations of soil nutrients [16, 17]. Based on the “regionalized variable” theory [18], geostatistical approaches such as ordinary kriging and cokriging have demonstrated to be useful for characterizing the spatial heterogeneity of soil properties. Although there are several limitations and disadvantages in kriging, for example, ordinary kriging requires that the data satisfy the stationary hypothesis [19] and exhibit normal or approximately normal distributions [20]. Kriging is easy to conduct without additional variables and can provide not only the best linear unbiased estimates for unsampled locations [21, 22] but also a measure of the uncertainty [20]. Consequently, soil nutrients have been estimated at different scales, from field to global by geostatistical techniques [16]. Studies at different scales have determined that the main factors influencing the spatial variability of SOC and STN were soil types [23], climate [24, 25], topography[26–28], land use patterns [29–31], tillage practices [1] and fertilizer application [32].

In recent years, numerous studies have been conducted in different areas of China including large-scale studies. Most studies of the spatial variations of soil nutrients, however, have focused on agro-ecological systems [21, 33–35]. There are relatively few reports in the red soil region of southern China, especially for areas with severe soil erosion. Soil erosion is still a major cause of land degradation and other environmental issues in China. High erosion rates can reduce soil thickness, alter soil hydrological properties, diminish soil productivity, and affect the potential for carbon sequestration and soil nutrient contents [36, 37]. Ecological reconstruction based on improving soil properties and increasing vegetation coverage is a promising approach for restoring soil productivity and sustainability [38]. Vegetation is closely related to soil nutrients and vegetation growth while community composition and tree species diversity all respond to soil nutrients. Many studies have demonstrated that soil quality is the key condition for ecological restoration [39]. Low soil fertility is an important biophysical constraint for vegetation restoration in degraded land. Therefore, understanding the spatial heterogeneity of soil nutrients is important for promoting vegetation recruitment and eco-rehabilitation.

Changting County is one of the most typical eroded soil regions in the hilly region of subtropical China and characterized by mountainous and varied topography. Due to poor soil fertility and flood hazards caused by soil erosion and less developed production technology, Changting County has been trapped in poverty and ecological degradation for a long period. In recent decades, various methods, such as closing hillsides for afforestation, planting trees and grass, fertilizing forests, and fruit cultivation, have been widely implemented to control soil erosion and mitigate ecological degradation [40–42]. These measures have achieved some success and serious soil erosion has been curbed and vegetation coverage has been significantly improved. As a model of soil erosion control in China, Changting's practices have been promoted as an example of ecological construction throughout the country. Numerous studies have been conducted at plot and catchment scales in Changting County [43–45] with erosion mechanisms [46, 47] and vegetation restoration modes [45] as the main research contents. However, no field study has yet been carried out to investigate the overall levels of SOC and STN in Changting County, which were inadequate in providing basic soil data to improve the soil based on local variability. In addition, this lack of information has led to a poor understanding of the spatial distributions of SOC and STN after years of treatment in Changting County. Thus, assessing the spatial variability of SOC and STN in the eroded hilly regions of subtropical China is urgent and is of great significance for effective soil restoration. We hope to fill this gap and provide a basis for soil erosion control and restoration in Changting County and even subtropical eroded areas. Therefore, considering the complex terrain and diverse land-use types of the southern red soil hilly region, our investigation was at a regional scale, variability of SOC and STN were analyzed in relation to topographic factors (e.g., elevation, slope steepness, slope aspect) and land- use type. The study can provide valuable information for understanding the spatial heterogeneity of soil properties in response to ecological restoration on a relatively large spatial scale.

The objectives of this work are to 1) estimate the contents of SOC and STN and investigate their spatial variability in an eroded hilly region located in southern China, as well as 2) assess the influence of topography and land-use type on SOC and STN.

## Materials and methods

### Ethics statement

This article does not contain any studies with human participants or animals performed by any of the authors.The soil survey in this study was carried out in the field and did not involve private land or nature reserve land, so no permits were required. The field study did not involve endangered or protected species.

### Study site

**Fig 1:**
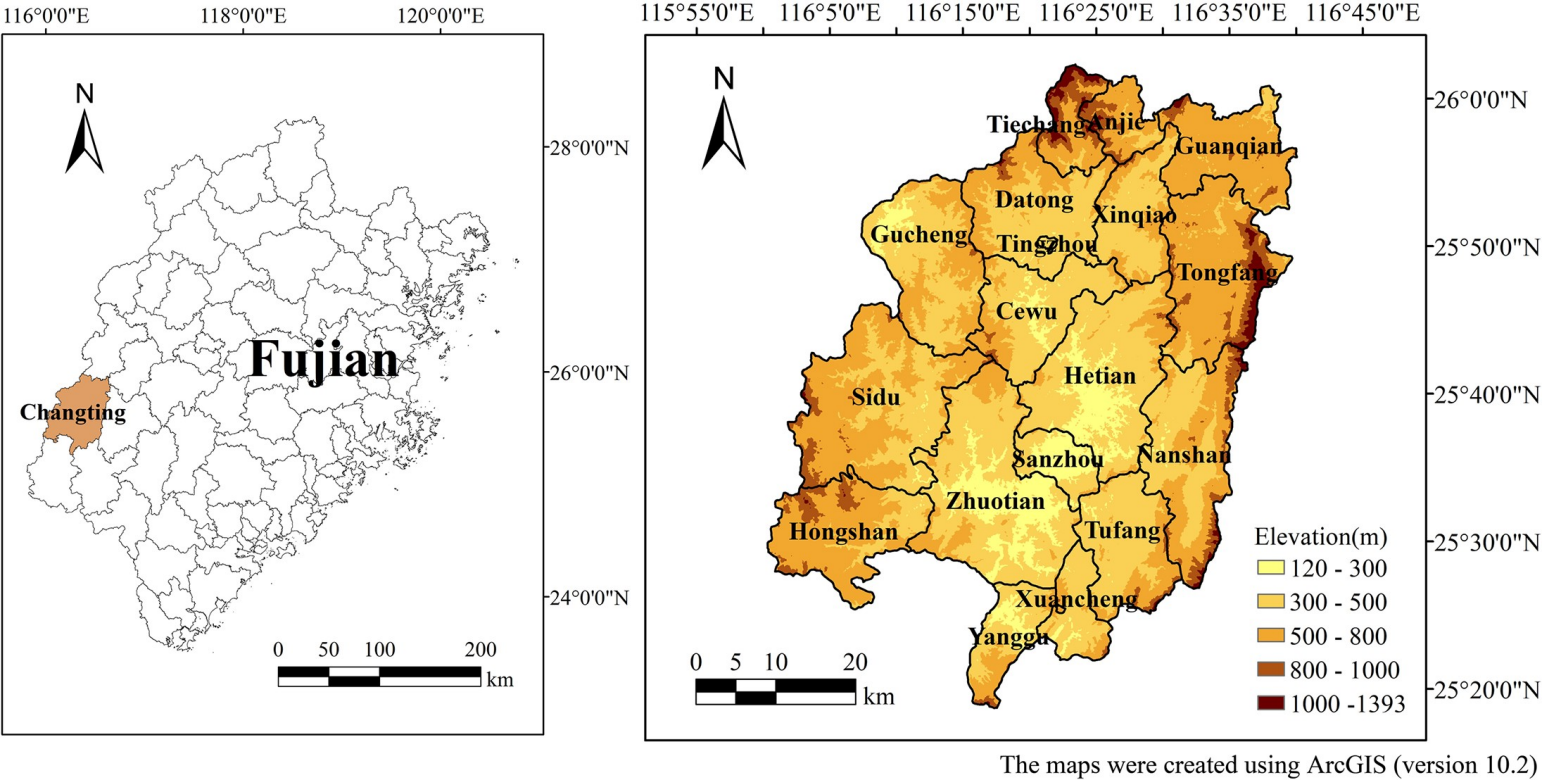
Location and elevation of Changting County, Fujian.

The study was conducted in Changting County, which covers an area of 3099 km^2^ and is located in the western Fujian Province of southeastern China (25°18′40″N-26°02′05″N, 116°00′45″E-116°39′20″E). Changting County is located at the southern part of the WuYi Mountains. The elevations range from 120 to 1,393 m above mean sea level (Fig 1). The geomorphology consists of low mountains, hills and terraces. The region has a subtropical humid monsoon climate with dry (October to February) and wet (March to September) seasons. The mean annual air temperature is 18.3°C, and the average annual precipitation is 1,730 mm. The principal soil type in the region is red soil derived from granite with high erodibility [48], which is a highly erosion-prone soil that is susceptible to water erosion. The zonal vegetation type is a subtropical evergreen broad-leaf forest. Owing to destruction caused by human activities, the native vegetation has been entirely replaced by secondary forests. The current dominant vegetation species is *Pinus massoniana* with *Dicranopteris dichotoma*. Soil and water loss in *Pinus massoniana* woodland is serious due to simple layer structure and sparse understory vegetation. Most of the land in this area is woodland with small amounts of farmland.

### Sampling strategy and soil analysis

**Fig 2:**
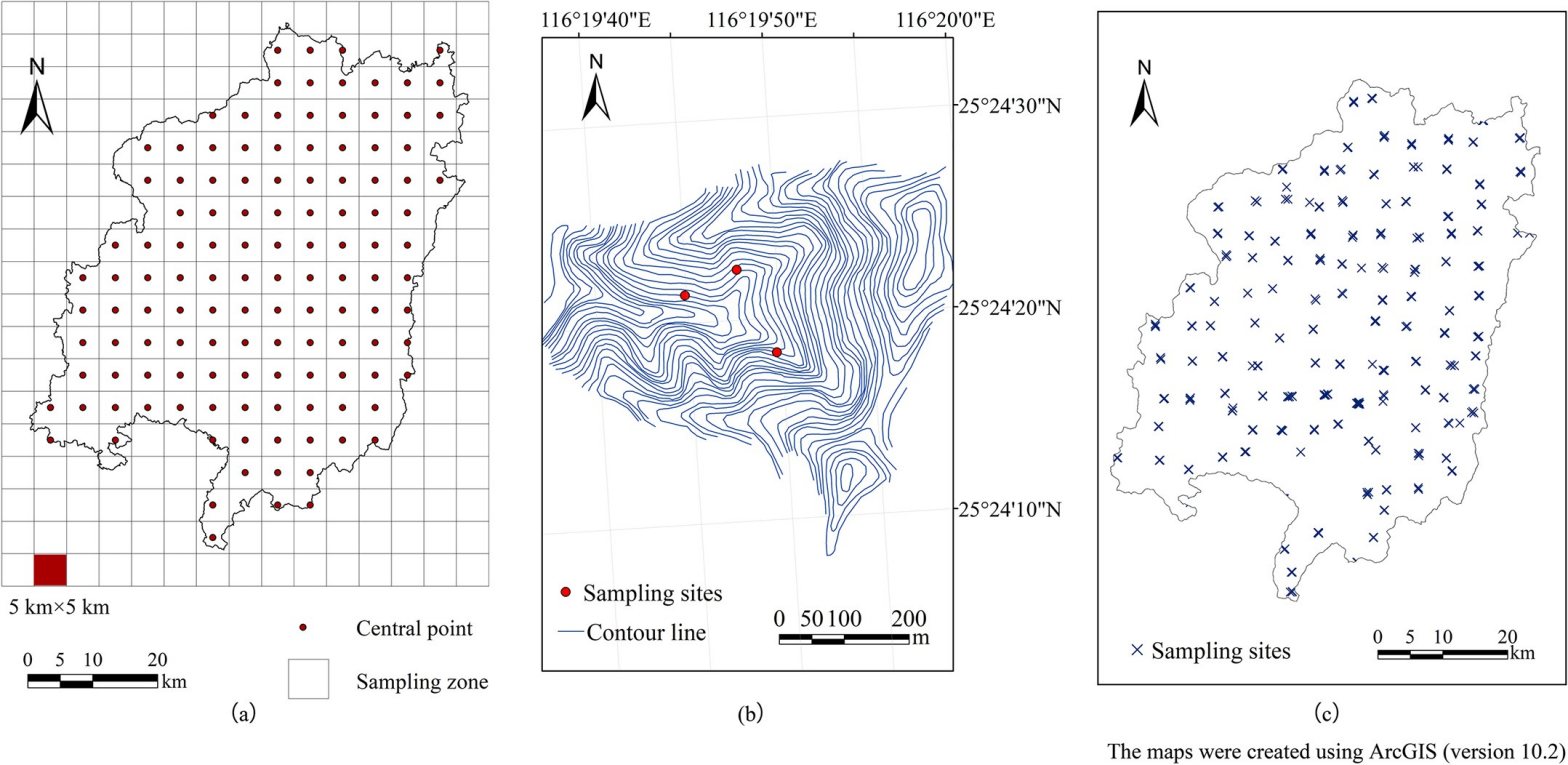
Sample design and distribution of sampling sites in Changting County.

The sampling strategy for this study was based on the field survey unit design in soil and water conservation survey (the first national water conservancy survey of China) [49]. Following the principles of uniform distribution and random sampling, the sampling zones were defined on a grid (approximately 5 km×5 km) for all of Changting County using a stratified sampling approach. A total of 125 sampling zones and their central point distribution map were obtained by using ArcGIS (version 10.2) (Fig 2A).

By referring to the 1:10000 topographic map, a small watershed with an area of 0.25–1 km^2^ near the center of each sampling zone was selected as the survey object and followed the principle of complete landforms and ensured good accessibility while avoiding as many artificial buildings as possible. Considering the land classifications and complex terrain of the southern red soil hilly region, three sample points were selected at different positions in each small watershed (Fig 2B). In total, 375 mixed topsoil samples (0-20cm) were collected from the selected sites in October 2008 as shown in Fig 2C. The figures were all created using ArcGIS (version 10.2) software.

After air drying, roots and rock fragments were removed from the samples, which were then ground and sieved through a 0.25-mm sieve and were finally homogenized. The soil samples were analyzed in the State Key Laboratory of Subtropical Mountain Ecology (Ministry of Science and Technology and Fujian Province funded). SOC was measured following the potassium dichromate volumetric method, and STN was analyzed with the Kjeldahl procedure [50]. A handheld GPS was used to accurately record the latitude and longitude of each sampling site. The topographical parameters (e.g., elevation, slope and slope aspect) were extracted from the 30m-DEM by ArcGIS. Elevation and slope were divided into three and five groups, respectively, and the slope aspect was classified into four types.

### Statistical and geostatistical analysis

Descriptive statistics for SOC and STN were calculated using IBM SPSS (Version 21.0). The basic statistics calculated included the minimum, maximum, mean value, standard deviation, coefficient of variation, skewness, and kurtosis. Pearson correlation coefficients (two-tailed) were computed for SOC, STN, and topographical parameters to create a correlation coefficient matrix. The normal distributions of the data were estimated based on the skewness, kurtosis, and Kolmogorov-Smirnov (K-S) test. The coefficient of variation (*CV*) is a vital and widely used dimensionless index that can indicate the overall variability of variables. Based on the classification proposed by Nielsen and Bouma [51], *CV* ≤ 10%, 10% < *CV* ≤ 100%, and *CV >* 100% indicated weak, moderate, and strong variability, respectively.

Outliers usually refer to extreme values that naturally exist in the study area or that are caused by errors in sampling and laboratory analysis [44, 52]. The outliers in the range have a great influence on the accuracy of the semivariogram theoretical model [53]. They mainly affect the nugget (*C*_0_). The nugget (*C*_0_) increases with more outliers and the influence of random components is strengthened while the influence of spatial autocorrelation is weakened [53]. Outliers can cause distortions that violate geostatistics [54] and cause the variograms to be unstable [55]. The threshold method is a simple and effective method for identifying specific outliers [52]. Data greater than or less than three standard deviations from the mean (σ±3s) are identified as outliers where σ and s represent the mean value and standard deviation, respectively [44, 56]. For kriging analysis, the extreme data can be removed and replaced with the normal maximum and minimum values [56].

Pearson correlation analysis and regression analysis assessed the relationships of SOC and STN with topographical factors. Analysis of variance (ANOVA) and least significant difference (LSD) tests were performed to determine the significant differences between topographical factors and land-use types on the SOC and STN concentrations. Differences were considered to be statistically significant at the 0.05 level. All statistical analyses were calculated using IBM SPSS (Version 21.0).

Descriptive statistical methods assume that variables are completely independent or without spatial structure [35].Traditional statistics can only describe average changes in spatial variability. In geostatistics, spatial variation is considered to be random and is modeled through a stochastic process. Additionally, the geostatistical approach is based on the hypothesis that points are located closer together in the space share more similar values than those farther apart [57]. Therefore, geostatistical analysis was conducted to quantify specific spatial patterns for mapping SOC and STN levels in the study area.

The semivariogram and kriging methods have been acknowledged as the main spatial interpolation techniques among the geostatistical methods. The semivariogram technique is used to quantify the spatial variability of a regionalized variable and provides information regarding the input parameters for the kriging interpolation method [18, 58].

Semivariogram can be expressed as follows:

$$\gamma (h)=\frac{1}{2N(h)}{\sum_{i=1}^{N(h)}\left[Z(xi)‐Z(xi+h)\right]}^{2}$$ (1)

where *γ*(*h*) represents the semivariance at a given lag distance (*h*), *Z*(*xi*) is the measured value for variable *Z* at the location of *xi*, and *N*(*h*) is the number of sample points pairs separated by *h*. The semivariogram mainly depends on the sampling interval and the measured data.

Several experimental semivariogram theoretical models are generally used for fitting in soil studies, i.e., the spherical, exponential, and Gaussian models [59]. The models were selected based on the coefficient of determination (*R*^2^) and the residual sum of squares (*RSS*). The best-fitted model was that with the largest *R*^2^ and lowest *RSS* [35], and provides the model provided information and input parameters of the spatial structure for interpolation [10]. *γ*(*h*) increases with increasing distance; the minimum distance at which the semivariogram reaches a steady state is the range, and shows that the samples are considered spatially independent. Sill (*C*_0_+ *C*) is the maximum semi-variance value that corresponds to the range. Nugget (*C*_0_) is the variance at 0 lag distance, caused by measurement errors and internal variance at a smaller scale than that of the sampling strategy [60]. The nugget/sill ratio has been used extensively to assess the spatial dependency of different variables. Ratios of < 25%, 25%-75%, and > 75%, indicate that the variable has strong, moderate, and weak spatial dependence respectively. A strong spatial dependence is normally caused by inherent factors, while weak spatial dependence is attributed to extrinsic factors [9].

The kriging method is the second step of geostatistical analysis. Ordinary kriging is one of the most common and optimal interpolating methods of spatial prediction that can provide an unbiased estimation for unsampled locations and reduce the impact of outliers [21, 22]. Both the distance and degree of variation between known points were considered when predicting the values of unknown points [61]. Ordinary kriging calculates the *Z* value at point *x*_0_ by the formulae:

$$Z^{\prime }(x_{0})=\sum_{i=1}^{n}\lambda_{i}Z(x_{i})$$ (2)

where *Z*′(*x*_0_) is the estimated value at unknown points and *λ_i_* is the weight used for each of the *i* neighboring samples *Z*(*x_i_*) [62].

The cross-validation procedure was followed to evaluate the prediction efficiency of a model. In this approach, the errors between predicted and observed values are obtained by taking each observation out of the sample pool in turn and estimating the errors using those that remain. The performance of a model is validated by the errors and the best model is one with the following properties: the mean error (ME) should ideally be 0, mean squared error (MSE) should approach 0, root-mean-square error (RMSE) should be the lowest, average standard error (ASE) and the root-mean-square error (RMSE) are the closest, and root-mean-square standardized error (RMSSE) should approach 1.

Semivariogram analysis of SOC and STN was conducted using GS+ (version 9.0), while spatial distribution maps were created using ArcGIS (version 10.2).

## Results

### Descriptive statistics

The descriptive statistical parameters that are usually recognized as indicators of the central trend of the data [32] are analyzed in Table 1. The SOC concentrations ranged from 0.07 to 56.86 g kg^-1^ with a mean of 15.85g kg^-1^, and those of STN ranged from 0.03 to 3.50 g kg^-1^ with a mean of 0.98 g kg^-1^. According to the skewness, kurtosis, and K-S test, the original SOC and STN data were not normally distributed (*P* < 0.05). After log-transforming the data, STN followed a normal distribution, while the SOC content still did not pass the K-S test. However, the log-transformed SOC data were close to a normal distribution with relatively small skewness and kurtosis. Semivariance analysis usually requires data with a normal or approximately normal distributions. Therefore, the log-transformed data of SOC and STN were used for all of the following analyses. In standard statistical analyses, the coefficient of variation (*CV*) is the most discriminating indicator for revealing heterogeneity. The *CV* values of SOC and STN in our study area were 72.73 and 74.61, respectively, which indicated moderate variation. The threshold method was used to recognize outliers and showed that SOC and STN contained four and one outliers, respectively, which would cause distortion that violates geostatistical theory [63] and cause the variogram to be unstable [55]. For kriging analysis, the extreme data were replaced with the normal maximum and minimum values.

**Table 1:** Descriptive statistics of SOC and STN in topsoil.

Items	Samples	DT^a^	Min	Max	Mean	SD^b^	Skewness	Kurtosis	K-S *P* value	*CV*^c^
(g kg^-1^)										(%)
SOC	375	S	0.07	56.86	15.85	11.53	-0.50	-0.47	0.004	72.73
STN	375	LN	0.03	3.50	0.98	0.73	0.22	-0.87	0.097	74.61

^a^ Distribution type: S is skewed distribution, LN is log-normal distribution.

^b^ Standard deviation.

^c^ Coefficient of variation.

### Geostatistical analysis

#### Semivariogram and spatial structure of SOC and STN

**Fig 3:**
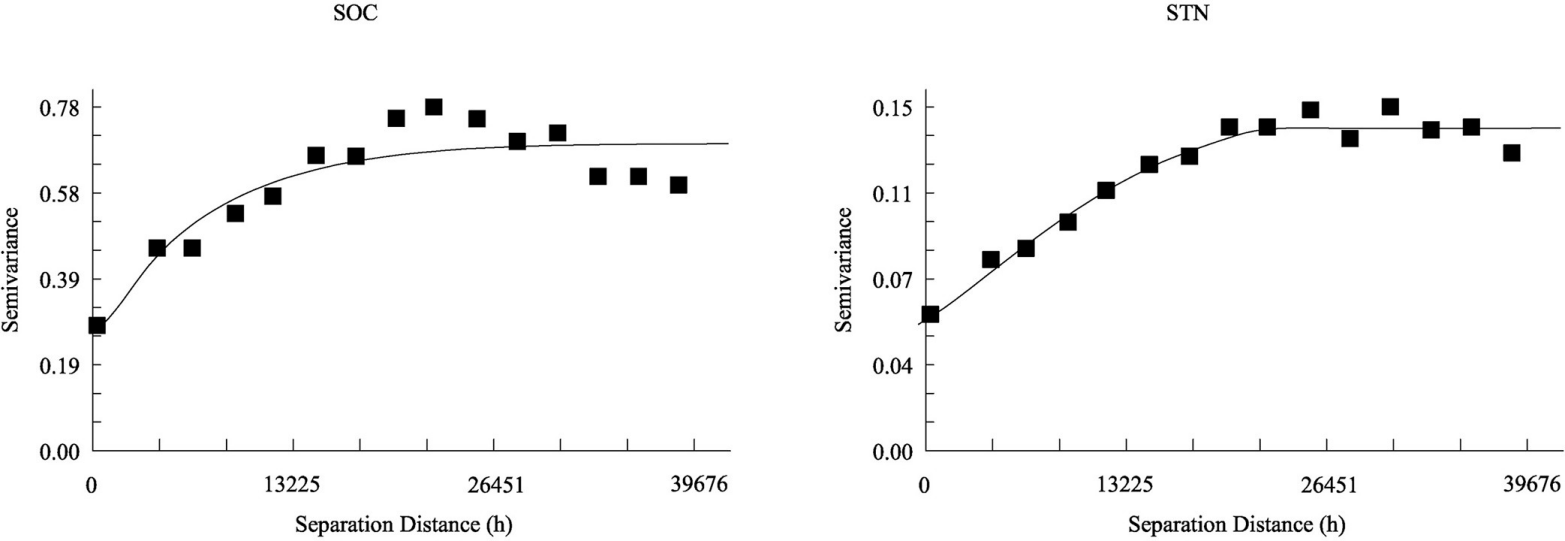
Semivariogram (dots) and fitted models (lines) of SOC and STN.

The semivariogram model and parameters of the best-fit models for SOC and STN are shown in Table 2 and Fig 3. The exponential model was the most suitable model for revealing the spatial distribution of SOC based on the highest *R*^2^ and lowest *RSS* values while STN was fitted best by the spherical model. The models showed that spatial correlation decreased with increasing distance before reaching the range (Fig 3). Both SOC and STN exhibited positive nugget (*C*_0_) values of 0.228 and 0.055, respectively. The sill (*C*_*0*_*+C*) indicates the total spatial variability within a system. SOC and STN exhibited moderate spatial dependence, with nugget/sill ratios of 32.62 and 39.83, respectively. The spatial range of SOC and STN were approximate and were much larger than the sampling interval (5 km), which indicated that the sampling approach in our study was suitable for detecting the spatial variability of soil properties, and ensured the precision of interpolation [64].

**Table 2:** Parameters for the SOC and STN semivariogram models.

Parameter	Model	Nugget (*C*_*0*_)	Sill (*C*_*0*_*+C*)	Nugget/Sill (%)	Class	Range(km)	*R*^*2*^	*RSS*
SOC (g kg^-1^)	Exponential	0.228	0.699	32.62	moderate	20.76	0.811	0.050
STN (g kg^-1^)	Spherical	0.055	0.138	39.83	moderate	24.16	0.966	0.006

#### Spatial distribution of SOC and STN

**Fig 4:**
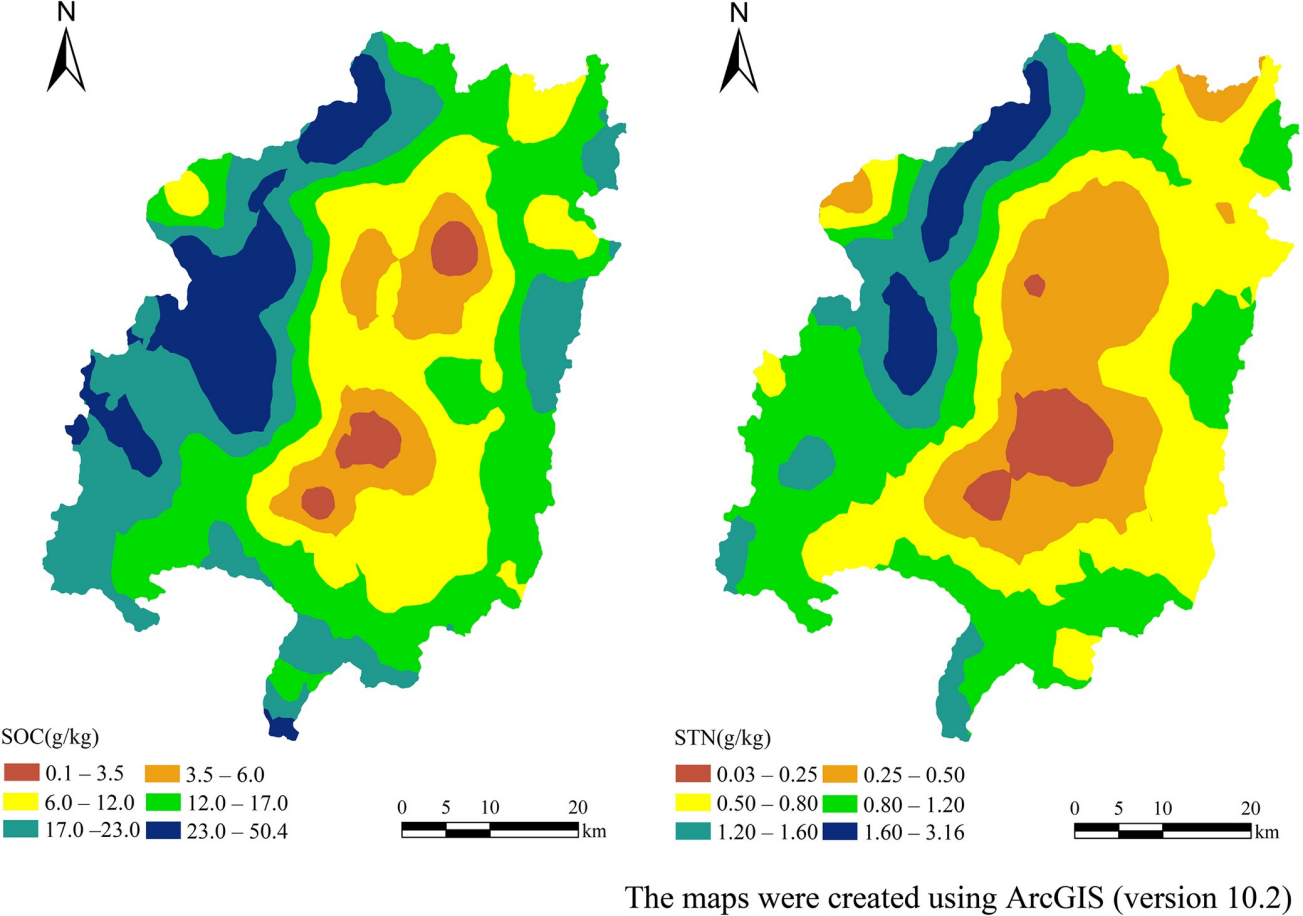
Spatial distribution maps of SOC and STN.

The ordinary kriging procedure in ArcGIS (version 10.2) was applied to calculate the values of un-sampled points using the parameters derived from the semivariogram models. The spatial distribution maps for SOC and STN are displayed in Fig 4. According to the China National Soil Survey conducted in the 1980s, the SOC and STN values were classified into six classes with ranges of > 23.0, 17.0–23.0, 12.0–17.0, 6.0–12.0, 3.5–6.0, and < 3.5, and > 2.0, 1.5–2.0, 1.0–1.5, 0.75–1.0, 0.5–0.75, and < 0.25, respectively.

The interpolated SOC values gradually decreased from the outside of the study area to its center (Fig 4A) and the spatial distribution pattern corresponded to the topography of Changting County. The low values of the fourth, fifth and sixth SOC- classes appeared in the center of the county and occupied 1,134.11 km^2^, or 36.60%, of the total land area (Table 3). This area mainly includes the towns of Xinqiao, Hetian, Cewu, Tingzhou, Zhuotian, Tufang and Sanzhou. The highest values for the first class were mainly distributed in Gucheng Town, part of Sidu Town and the area where Tiechang and Datong Towns came together and covered 358.68 km^2^, or 11.57%, of the total land area (Table 3).

**Table 3:** Areas and percentages of soil organic carbon (SOC) and total nitrogen (STN) by different classes in Changting County.

Class	1	2	3	4	5	6
SOC (g kg^-1^)	> 23.0	17.0–23.0	12.0–17.0	6.0–12.0	3.6–6.0	< 3.5
Area (km^2^)	358.68	697.11	909.10	781.81	274.05	78.25
Percentage (%)	11.57	22.49	29.34	25.23	8.84	2.53
STN (g kg^-1^)	>2.0	1.5–2	1–1.5	0.75–1	0.5–0.75	<0.5
Area (km^2^)	8.20	223.05	671.05	707.62	724.43	764.64
Percentage (%)	0.26	7.20	21.65	22.83	23.38	24.67

Most soil nitrogen is stored in organic matter [65] and total nitrogen and soil organic carbon are largely related to soil organic matter accumulations [66]. Pearson’s product moment correlation coefficients were calculated to characterize the relationships between SOC and STN (Table 3) and the results indicated a significant positive correlation between SOC and STN.

**Fig 5:**
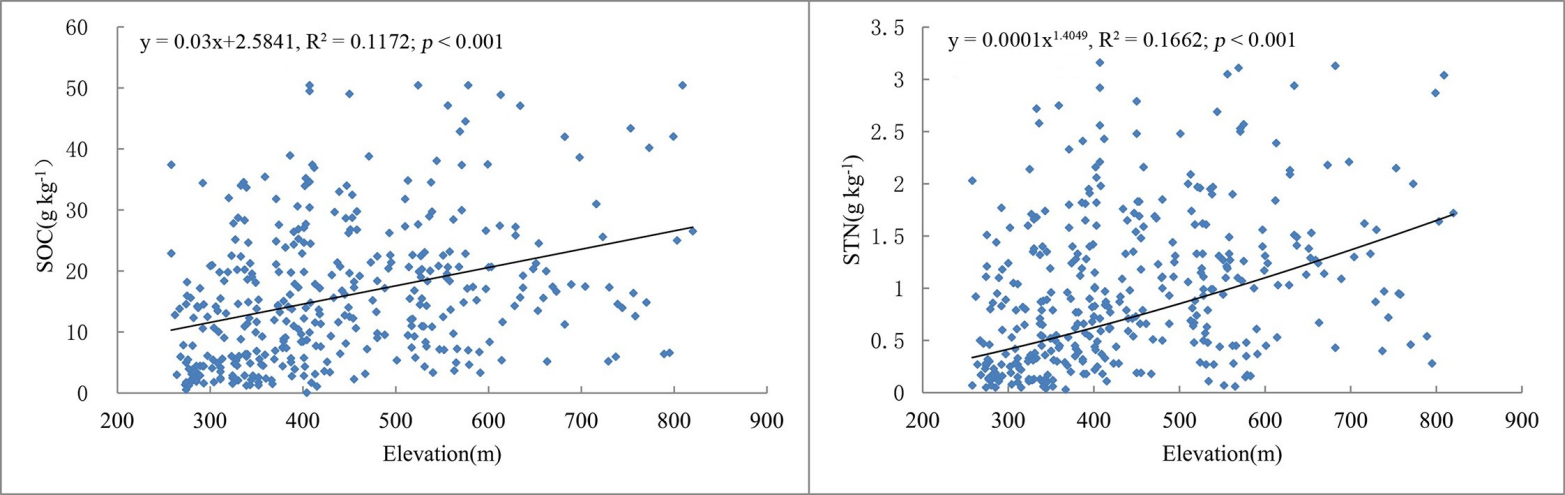
Regression analyses between SOC, STN, and elevation using scatter plots.

The spatial distribution trend of STN was similar to that of SOC as it declined gradually from the outside part of Changting County to its center. The soils had contiguous areas in the center of the county with relatively low STN contents below 0.50g kg^-1^ (Fig 5B) and these areas included part of Guanqian Town and the towns of Xinqiao, Tingzhou, Cewu, Hetian, Zhuotian, and Sanzhou. The areas of fourth, fifth and sixth STN- class land were 2,196.70 km^2^ in total and occupied 70.88% of the total area, and the sixth STN- class land covered the largest area of 764.64 km^2^ (24.67% of the county) (Table 3). The first-class STN land was mainly distributed in the north and west parts of the county and covered only 8.20 km^2^, or 0.26%, of the county (Table 3). In general, the STN values in the study area were low.

### Factors influencing the spatial variation of SOC and STN

#### Topographical factors

The landforms in the study area are mainly characterized by low mountains and hills, with elevations ranging from 120 to 1,393 m (Fig 1). The western part of the county is dominated by low mountains while the eastern and northern parts of the county are occupied by middle-sized mountains that descend from north to south.

Regression analyses with scatter plots were conducted to determine whether linear relationships existed between SOC, STN, and elevation (Fig 5) and these analyses confirmed that both SOC and STN increased with elevation. The SOC and STN values all showed a tendency of increasing with elevation.

The samples were stratified into three elevation groups, e.g., < 300 m, 300–500 m, and > 500 m and were based on the closest contour line to a sampling site. In the medium- and high-elevation mountains higher than 500 m, the mean values of the SOC and STN concentrations were comparatively higher (20.02 and 1.25 g kg^-1^, respectively) than those of the other two groups (Table 4). There was a significant positive correlation (*P* = 0.01) between SOC and STN (Table 5) and similar trends were observed between SOC, STN, and elevation.

**Table 4:** Comparison of the means of topsoil SOC and STN concentrations of different elevation groups.

Elevation (m)	n	Mean SOC(g kg^-1^)	Mean STN(g kg^-1^)
> 500	120	20.02 a	1.25 a
300–500	208	15.02 b	0.92 b
< 300	47	8.63 c	0.53 c
F		19.77**	20.23**

Different letters in the same column indicate significant differences at *P* < 0.01.

** *P* < 0.01.

**Table 5:** Bivariate correlations between the studied variables.

	SOC	STN
SOC	1	
STN	.824**	1
Elevation	.342**	.355**
Slope	.181**	.215**
Slope aspect	-0.072	-0.057

** Correlation is significant at the 0.01 level (2-tailed).

Analysis of variance was conducted for the SOC and STN concentrations of all topographical zones and the results showed that there were significant differences between the three groups in Changting County (FSOC = 19.77, *P* < 0.01; FSTN = 20.23, *P* < 0.01). Similar to the regression analyses, both SOC and STN increased with elevation. The K–S test indicated that all three groups passed the normality test (*P* > 0.05).

Based on the actual topography of Changting County, slope was categorized into five classes: flat (< 5°), gentle (5–8°), ramp (8–15°), abrupt (15–25°), and steep slope (> 25°).

**Fig 6:**
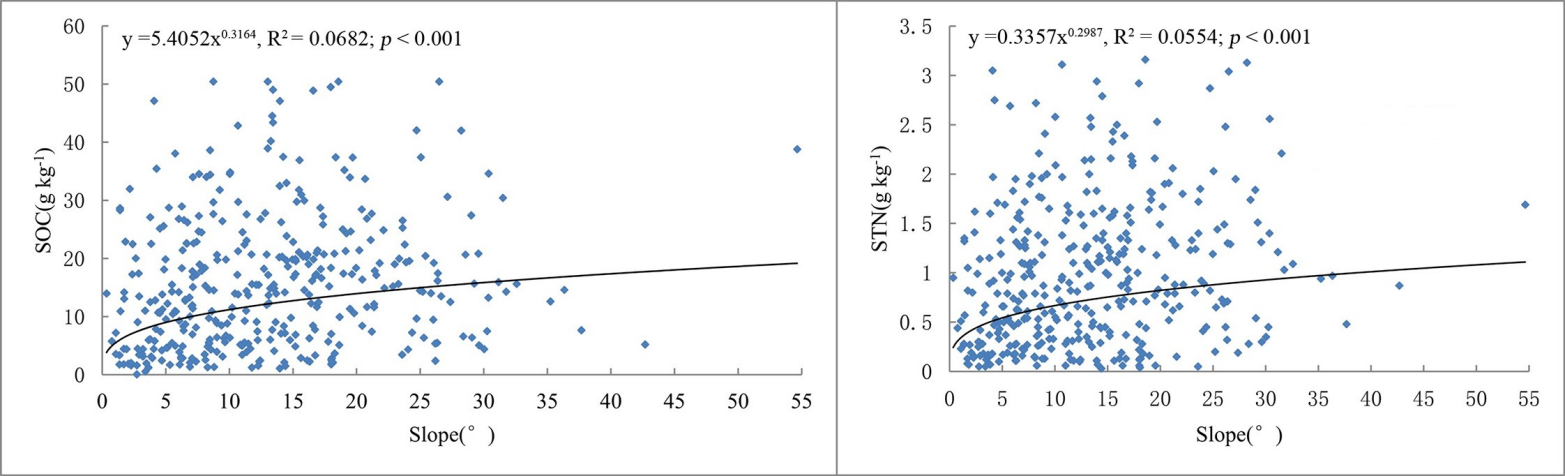
Regression analysis between the SOC, STN and slope using scatter plots.

Pearson’s product moment correlation coefficients were calculated to characterize the relationships between slope and SOC or STN (Table 5). The results suggested that slope was strongly positively correlated (at *P* = 0.01) with SOC and STN (Table 6). The correlations between SOC, STN, and slope were studied through scatter plots and regression analyses (Fig 6). SOC contents increased as the slope increased and reached a peak value at 15–25° and then decreased as the gradient continued to increase. However, STN contents increased gradually as the slope increased. The mean SOC and STN concentrations for different slopes are presented in Table 6. The SOC content for the 15–25° group and STN content for the > 25° group were relatively high, with mean values of 18.23 and 1.21 g kg^-1^, respectively.

**Table 6:** Comparison of the mean topsoil SOC and STN concentrations of different slope groups.

Slope (°)	n	Mean SOC(g kg^-1^)	Mean STN(g kg^-1^)
> 25	37	17.16 ab	1.21 a
15–25	103	18.23 a	1.12 a
8–15	116	16.41 ab	0.98 ab
5–8	59	14.16 b	0.88 b
< 5	60	11.33 b	0.67 b
F		4.11**	5.07**

Different letters in the same column indicate significant difference at *P* < 0.01.

** *P* < 0.01.

According to the variance analysis results (Table 6), there were significant differences between the SOM and STN of the five slope groups (F_SOC_ = 3.74, *P* < 0.01; F_STN_ = 5.21, *P* < 0.01). The K–S test indicated that all the five groups passed the normality test (*P* > 0.05).

Four slope aspects, e.g., sunny (157.5°-202.5°), semisunny (202.5°-337.5°), semishady (22.5°-157.5°), and shady (337.5°-22.5°) were selected to study the influence of slope aspect on soil properties.

**Fig 7:**
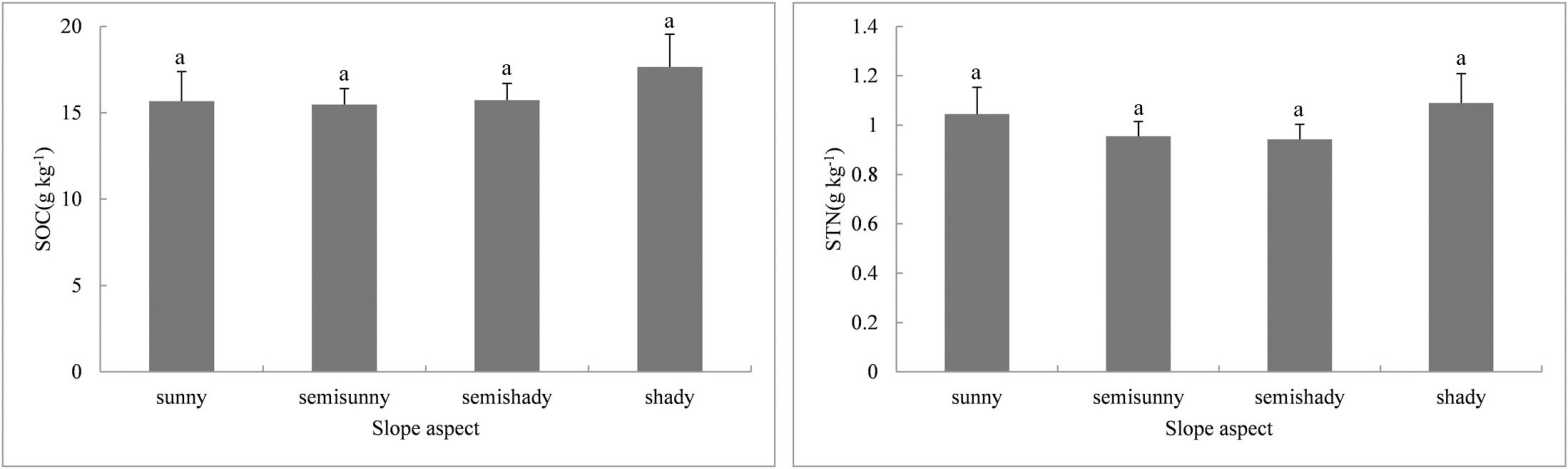
Comparison of SOC and STN concentrations among four slope aspects.

Pearson correlations were calculated to analyze the relationships between slope aspect, SOC, and STN (Table 5), and showed that the slope aspect response to SOC or STN was not significant. The mean SOC and STN concentrations under different slope directions are shown in Fig 7. As the slope direction changed, the order of SOC content decreased as follows: shady slope > semishady slope > sunny slope > semisunny slope, and the STN content decreased in the following order: shady slope > sunny slope > semisunny slope > semishady slope. Similar to Wang et al. [67], although the SOC and STN values in shady slope areas were higher than those in sunny slope areas, the results of variance analysis found no statistically significant variations in the SOC and STN concentrations between different slope directions in the study area (F_SOC_ = 0.385, *P* > 0.05; F_STN_ = 0.582, *P* > 0.05). The K–S test indicated that all four groups passed the normality test (*P* > 0.05).

Error bars represent the standard error of the means of SOC and STN at different slope aspects (e.g., sunny, semisunny, semishady and shady slopes). Same letters above columns indicate no significant difference at *P* > 0.05.

#### Land-use types

The land-use types for our samples were mainly forestland, farmland, garden land and grassland. Among them, forestland is the dominant land-use type in Chanting County and accounts for more than 70% of the total land area. Forestland in the county consist mainly of arbor trees. Farmland and garden land are mainly distributed in the mountainous basin valleys. In particular, most of the cultivated land is concentrated in the basin in the middle of the county. Grassland is scattered in the mountains and is mostly located in the western part of the county.

SOC under four land-use types followed the order of forestland > grassland > farmland > garden land and the STN concentrations were in the descending order of farmland > forestland > grassland > garden land (Table 7). However, no significant differences in SOC and STN were found between the four land-use types (FSOC = 1.23, P > 0.05; FSTN = 0.88, P > 0.05), which indicated that land-use had no significant influence on SOC and STN in our study.

**Table 7:** Statistical results of the topsoil SOC and STN concentrations under different land use types.

Land use types	n	Mean SOC(g kg^-1^)	Mean STN(g kg^-1^)
forestland	291	16.31 a	0.97 a
grassland	28	15.29 a	0.95 a
farmland	43	14.70 a	1.08 a
garden land	13	10.58 a	0.71 a
F		1.23	0.88

Same letters in the same column indicate no significant difference at *P* > 0.05.

## Discussion

### Spatial distribution characteristics of SOC and STN

The statistical analyses revealed that the variability of SOC and STN in the eroded hilly region was moderate which is in accordance with the reports of previous studies at different scales [34, 68]. Both SOC and STN exhibited positive nuggets (*C*_0_) which may have been caused by stochastic factors, small-scale variance, undetectable experimental error, or the existence of outliers [54]. In this study, experimental errors were minimized and outliers were removed. Thus, the nugget effect was most likely attributable to stochastic factors (such as human activity) and small-scale variations in some areas of the current sampling interval. For the nugget/sill ratios, SOC and STN exhibited moderate spatial dependence, which suggested that the spatial dependence of SOC and STN may both be attributed to intrinsic factors such as soil type, soil texture, soil formation and extrinsic variations such as land use, production management, and fertilizer application. Many previous studies have shown that the spatial variability of SOC and STN at large scales is mainly affected by climate, soil type, topography and land- use patterns [24, 25, 27–29, 31, 32]. Therefore, based on the characteristics of the study area, the effects of topography and land use on SOC and STN will be discussed further here.

According to the kriging-interpolated map, the entire study area was characterized by moderate or low concentrations of SOC and STN. SOC and STN exhibited relatively uniform distributions that decreased gradually from the outer part of the study area to the central part. Contiguous areas with low SOC and STN values in the center of Changting County mainly included the towns of Xinqiao, Hotan, Cewu, Zhuotian and Sanzhou. These low values could be attributed to the loss of topsoil SOC due to erosion. Influenced by landforms, the low mountains and hilly areas in the middle of Changting County overlap with the north-south and east-west transportation networks. Intensive human activities in this area have caused this area to have the most serious erosion. According to the soil nutrient grading standard of the second China National Soil Survey, the SOC and STN contents in the study area were still at moderate and low levels after years of erosion control, which suggests that soil nutrient improvement is a slow process.

### Effects of topography and land use on the spatial distribution of SOC and STN

The SOC and STN contents all exhibited large spatial variations along the elevation gradient in our study area. The results agree with those of Hu et al. [34] who found that SOM and STN concentrations increased with elevation at three spatial scales in suburban Beijing. Prietzel and Christophel [69] further found that the SOC at sites with high- elevations in the German Alps was especially high due to the low temperatures and high precipitation. The distribution of soil organic carbon at different altitudes may be attributed to changes in temperature [70], because low temperatures can aid the accumulation of organic matter. SOC content is mainly affected by the secondary effects of organic carbon oxidation, decomposition and transformation [71–73] and mineralization/accumulation rates [74–77]. As many studies have found [27, 78], with decreases in temperature, SOC and STN accumulate at higher elevations. However, both air temperature and soil layer thickness decrease with increasing altitude, which will also results in the change of vegetation distribution and productivity under different altitude gradients [79]. Therefore, increasing altitude may lead to less litter accumulation and low input of organic carbon and nitrogen in soil [80]. Sheikh et al. [80] found that the stocks of SOC decreased with altitude in the broadleaf temperate and coniferous subtropical forests of Himalaya zones. It indicates that the influence of altitude on SOC and STN content may depend on vegetation composition and climate change along altitude gradient [78]. In addition, erosion has a significant influence on soil properties in regions, which are characterized by undulating terrain. Topography is a major natural factor affecting soil erosion in hilly areas [81, 82]. According to the statistics, most of the soil erosion in Changting County is distributed in hilly areas with altitudes of 200–500 m and accounts for 82.14% of the total area of soil erosion [83]. Frequent human production activities and high utilization rates of forest and soil resources are found at the low and middle elevations where the majority of towns and villages are located. Orchards and slope farmland are also relatively concentrated in this zone so soil erosion is serious. Thus, SOC and STN contents may increase with elevation due to the reduction of soil erosion.

The SOM and STN concentrations were also significantly correlated with the slope. This result is consistent with the finding of Guo et al. [33], but differed from the conclusion of Wang et al. [78] who found that SOC and STN values on gentle slopes (< 10°) were higher than those on steeper slopes (> 25°). The variation characteristics of SOC and STN contents for different slopes are closely related to slope distribution patterns, land-use types and soil erosion intensities in the study area. The gentler areas in the middle of the study area are mainly inhabited by humans. Cultivated land and garden land are mostly distributed in the range of 6–25°, and the slope area of 15–35° is dominated by forestland. The luxuriant vegetation in the flat and gentle slope areas generally allows accumulation of soil organic matter. The mountain vegetation in Changting County, however, was severely damaged due to social and historical factors (i.e., the scramble for trees caused by disputes over forest rights among clans, large numbers of trees were cut down for military use in war and lumbering for fuelwood in poor regions). Therefore, the original vegetation in the gentle areas has been completely destroyed and is now mainly secondary vegetation, while the vegetation on steep slopes is well preserved. Slope is an important soil erosion factor, and soil degradation due to erosion is considered to be inextricably linked to the loss of SOC [84, 85]. Changting County has been severely eroded for many years. Slope steepness affects the intensity of soil erosion by controlling the rate of soil redistribution across hill slopes [21]. In theory, greater slopes may result in higher risks of runoff and soil loss [21, 86, 87]. However, with the development of the economy in Changting, land development was intensive in flat and gentle regions, consumption of forest resources increased rapidly, and thus led to serious erosion. As the population increased rapidly, moderate slope areas were developed into planting orchards. However, in the process of development and utilization, nonstandard engineering measures and low amounts of ground cover resulted in increased soil erosion. Therefore, the frequent human activity in low and moderate slope areas led to more serious erosion and declines in SOC and STN. In contrast, the preservation of vegetation in high slope areas with little human interference was better, and the SOC and STN contents were higher.

As the slope aspect changed, the data also indicated that the SOC and STN contents on shady slopes were relatively higher than those of sunny slopes, which is consistent with the results of some previous studies [27, 88–90]. According to Wang et al. [78], in subalpine forested catchment soils, the mean SOC and TN concentrations on shaded and semishaded slopes were 31.2% and 24.6% higher than those on sunny and semisunny slopes, respectively. The reasons for this are as follows. With good hydrothermal conditions, evapotranspiration on shaded northern slopes is much lower than that of exposed slopes and therefore, soil moisture contents are relatively high throughout the year. The lush vegetation and abundance of surface biomass in shady slope areas become an important source of soil organic carbon. From shady to sunny slopes, light intensity increases, and vegetation transpiration and soil moisture evaporate faster, causing a significant loss of soil moisture and hindering SOC accumulation, which then affects the total soil nitrogen content.

The slope aspect affects the amount and intensity of solar radiation as well as the angle between the ground and wind direction [91]. Surface temperature, soil temperature, soil moisture, and vegetation vary greatly among different slope aspects. It is generally expected that soil nutrient mineralization varies greatly between different slope aspects [92]. However, in this study, we did not observe any significant effects of slope aspect on SOC and STN. This result indicates that slope aspect is not a principal factor for the spatial variation of SOC and STN as human activities, such as land reclamation and cultivation, weaken the effect of slope direction.

In our study, the SOC and STN contents were higher in forestland, grassland and farmland but were lower in garden land and corroborated the findings of Dessalegn et al. [93]. Generally, forestland and grassland were less disturbed by human activities. For one thing, the large vegetation biomass, abundant land litter and relatively thick humus layer [94] in forestland affect SOC by increasing the input of soil organic matter. At the same time, it can protect the soil from the impact of erosion by reducing the loss of organic matter. High forest coverage, canopy shade and litter cover have impacts on soil hydrothermal conditions, slow the mineralization of soil organic matter, and thus facilitate the accumulation of soil carbon and nitrogen. The relatively high SOC in grassland might be due to the dominance of roots, humus and associated organisms. In farmland management, such measures as turning over soil and weeding change the soil temperature, humidity, porosity and other conditions, enhance soil microorganism activity, and accelerate the decomposition process of soil organic matter. The differences in soil organic carbon and total nitrogen contents between farmland and garden land may be correlated with fertilizer input. Generally, the amounts of organic fertilizer applied to farmland were relatively large and plant roots and stubble increased the input of soil organic materials after crop harvests while there was almost no input of other organic materials except for organic fertilizer in garden soil. Many studies have shown that different land-use patterns have significant effects on soil organic carbon and total nitrogen contents at large scales [30, 31, 34, 94] but such results were not observed in this study. These results may be because the land- use patterns in the study area had little influence on SOC and STN distribution or they might be due to the relatively greater number of samples in forestland than other land-use types by the systematic sampling conducted. Therefore, it is necessary to conduct further research on SOC and STN contents in this region through sampling at different scales and for different land-use types.

The results obtained in this research were derived only from the study of the impacts of topography and land-use type on SOC and STN in a hilly region. In fact, the influence of anthropogenic factors often changes due to regional policy interventions and natural factor changes. Future studies should focus on the combined effects of various influencing factors and suggest reasonable and scientific countermeasures for ecological restoration.

## Conclusion

The results reported in this study revealed that SOC and STN exhibit moderate variations, and that the entire study area is characterized by moderate or low concentrations of SOC and STN. A number of erosion control measures have been applied in Changting County and soil erosion has been initially restrained but the recovery of soil fertility including the increase of SOC and STN contents proceeds very slowly.

Both STN and SOC showed a spatial variation trend of gradually decreasing from the outside to the center of the study area, which is consistent with the topography of Changting County. The highest values were observed in the western and northern areas while the lowest SOC and STN levels were observed in the center of the study area with serious soil erosion, suggesting that soil nutrient improvement in degraded areas is a slow process. More effective measures are recommended for further ecological restoration in the central part of the country to improve soil nutrients. Our results show that topography plays an important role in controlling the spatial diversity of SOC and STN by influencing regional environmental conditions and soil erosion processes. The SOC and STN concentrations are positively correlated with elevation and slope. As the slope aspect changes, the SOC and STN contents on shady slopes are relatively higher than those of sunny slopes. The SOC and STN contents are higher in forestland, grassland and farmland but are lower in garden land. However, there are no significant variations between the SOC and STN for different slope directions and land-use types. Topography plays a greater role on SOC and STN levels than land-use types. Therefore, suitable soil erosion control measures should be adopted according to the different terrain characteristics in the hilly region for soil fertility recovery.

In addition, to improve our understanding of the spatial variability of SOC and STN in response to ecological restoration, the long-term spatiotemporal dynamics of SOC and STN should be examined in future studies.
